# Severe bilateral chorioretinopathy associated with ipilimumab in a patient with metastatic melanoma

**DOI:** 10.3205/oc000262

**Published:** 2025-11-25

**Authors:** Víctor Manuel Asensio-Sánchez

**Affiliations:** 1 Ophthalmology Department, University Clinical Hospital, Valladolid, Spain

**Keywords:** ipilimumab, metastatic melanoma, immune checkpoint inhibitors, central serous chorioretinopathy, retinal pigment epithelium detachment, ocular toxicity, vision loss

## Abstract

**Objective:**

To report a rare case of severe bilateral chorioretinopathy associated with ipilimumab in a patient with metastatic melanoma, highlighting the ocular toxicities that may arise from immune checkpoint inhibitors.

**Methods:**

A 38-year-old woman receiving ipilimumab (3 mg/kg every 3 weeks) for metastatic melanoma presented with painless bilateral vision loss following her third dose. Clinical assessment included visual acuity testing, fundus examination, fluorescein angiography (FA), and optical coherence tomography (OCT) to evaluate retinal changes. The ipilimumab treatment was discontinued, but the patient refused corticosteroid therapy.

**Results:**

Fundus examination and OCT revealed bilateral serous retinal detachments with retinal pigment epithelium (RPE) detachment. FA demonstrated multiple pinpoint leakage areas at the RPE level. Over a three-month follow-up period, visual acuity further declined, resulting in total vision loss in one eye and persistent bilateral serous detachments despite cessation of ipilimumab.

**Conclusions:**

This case highlights the potential for severe, irreversible vision loss due to ipilimumab-associated chorioretinopathy, underscoring the importance of early ophthalmological assessment and continuous monitoring in patients undergoing immune checkpoint inhibitor therapy. Early recognition and intervention are critical, especially when systemic corticosteroids are not administered.

## Introduction

Ipilimumab is a monoclonal antibody targeting cytotoxic T lymphocyte antigen-4 (CTLA-4), used in the treatment of advanced and unresectable cutaneous melanoma [1]. While it has shown significant benefits in overall survival, it is associated with immune-mediated adverse effects, including rare ocular events. We report the case of a woman who developed bilateral multifocal serous chorioretinopathy after receiving ipilimumab, emphasizing the importance of early recognition of these adverse effects in patients undergoing immunotherapy.

## Case description

**Figure 1:**
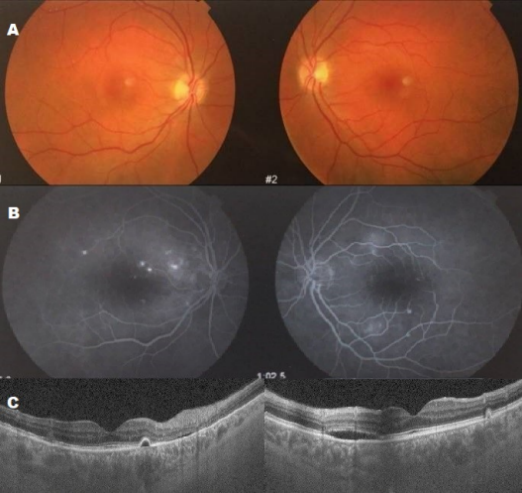
A: Fundus photographs showing multiple serous retinal detachments with retinal pigment epithelium (RPE) detachment in both eyes. B: Fluorescein angiography showing multifocal bilateral hyperfluorescent spots at the posterior pole with subretinal leakage and dye accumulation. C: OCT revealing hyporeflective areas beneath the retina, suggestive of subretinal fluid, associated with bilateral RPE detachment

A 38-year-old woman with metastatic melanoma was treated with ipilimumab (3 mg/kg every 3 weeks). After her third dose, she began experiencing painless bilateral vision loss. Visual acuity was 0.16 in the right eye (OD) and 0.20 in the left eye (OS). Fundus examination revealed multifocal bilateral serous retinal detachments (SRDs) (Figure 1A (Fig. 1)). Fluorescein angiography (FA) showed multiple areas of leakage (Figure 1B (Fig. 1)), and OCT confirmed serous detachments of the retinal pigment epithelium (RPE) (Figure 1C (Fig. 1)). There was no evidence of active intraocular inflammation in either eye.

**Figure 2:**
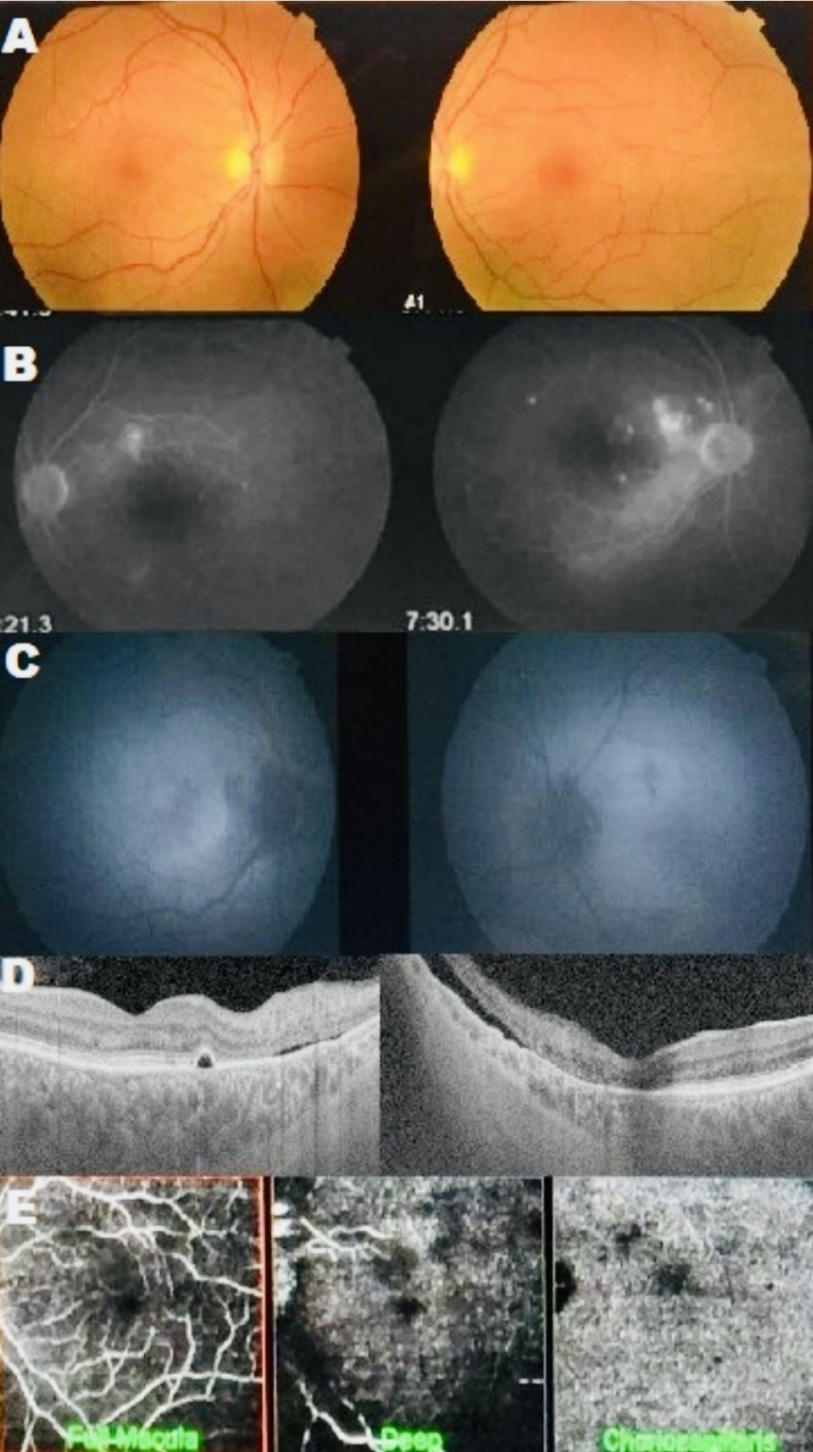
A: Fundus photographs showing serous retinal detachment (SRD) three months after ipilimumab discontinuation. B: Angiograms showing multiple leakage areas associated with increased background fluorescence. C: Autofluorescence images showing hypoautofluorescent areas with hyperautofluorescent zones corresponding to the SRD. D: OCT revealing multiple RPE detachments associated with SRD. E: Angio-OCT showing dark areas corresponding to SRD with abnormal and telangiectatic choroidal vessels

Indocyanine green angiography was not performed. Ipilimumab was discontinued, but the patient declined high-dose systemic corticosteroid therapy. Three months later, visual acuity in the OD had reduced to 0.1, and in the OS to hand movements. Studies showed persistent SRDs (Figure 2A, B (Fig. 2)) and choroidal changes with areas of hypo- and hyperautofluorescence (Figure 2C (Fig. 2)) on autofluorescence, and persistents SRDs (Figure 2D (Fig. 2)) and abnormal choroidal vessels on angiographic OCT (Figure 2E (Fig. 2)).

## Discussion

Since its approval in 2011, ipilimumab has become a cornerstone in the treatment of advanced melanoma, significantly improving survival in patients with metastatic melanoma and prolonging progression-free survival in responders [1], [2]. However, this benefit comes with a profile of immune-mediated adverse effects distinct from the typical side effects of conventional chemotherapy. By inhibiting CTLA-4, ipilimumab enhances immune activation, which can lead to inflammatory and autoimmune adverse events in various organs, including the eyes [3], [4]. Ocular toxicity is rare, reported in fewer than 1% of patients, but when it occurs, it can manifest as severe forms such as uveitis, scleritis, and, in exceptional cases, thyroid eye disease, optic neuritis, and multifocal serous chorioretinopathies [5], [6], [7]. In the present case, the patient developed bilateral multifocal serous chorioretinopathy after receiving the third dose of ipilimumab. Optical coherence tomography (OCT), fluorescein angiography, and autofluorescence confirmed the presence of SRDs and RPE detachment, along with choroidal vascular changes suggestive of ischemic endothelialopathy. The pathophysiology behind this clinical presentation is not fully understood, but several studies have proposed an immune-mediated mechanism in which disruption of the blood-retina barrier and cytokine activation may lead to an inflammatory vasculopathy similar to that observed in other tissues treated with ipilimumab [8]. There have been documented cases of ocular toxicity induced by ipilimumab that show similar characteristics to those observed in this patient. Wong et al. [7] described a case of Vogt-Koyanagi-Harada (VKH) syndrome induced by ipilimumab, which presented with serous detachments, although in that case, there was evidence of active intraocular inflammation, which distinguishes it from the clinical presentation here. Crews et al. [8] and Mantopoulos et al. [9] reported cases of choroidopathy and bilateral serous detachments associated with ipilimumab, suggesting either an autoimmune or ischemic pathogenic mechanism. The difference in our case is that the patient did not present active ocular inflammation or optic nerve hyperemia, pointing to a more vasculopathic and ischemic mechanism. This hypothesis is supported by the dark areas observed on angiographic OCT, which correspond to abnormal, telangiectatic choroidal vessels, suggesting microvascular damage possibly due to multifocal endothelialopathy [10]. It is noteworthy that despite the early discontinuation of ipilimumab, the clinical condition continued to worsen, with significant vision loss over the three-month follow-up period, consistent with previous reports of persistent or progressive toxicity even after drug withdrawal. Padda et al. [10] described a case of digital vasculitis associated with ipilimumab that led to amputations, reinforcing the idea that some adverse effects may be irreversible or continue to progress even after drug cessation. This case underscores the need to establish visual monitoring protocols for patients receiving ipilimumab, especially considering that treatment options to mitigate ocular toxicity are limited and not always accepted by patients, as seen in this case where the patient refused high-dose systemic corticosteroids. In situations where corticosteroids are unacceptable or contraindicated, alternative immunomodulatory therapies may be considered, although evidence of their effectiveness in such cases remains limited. This case highlights the importance of ophthalmologists being familiar with immune-mediated ocular adverse effects that may arise in patients treated with immunotherapy. In patients presenting with visual changes during ipilimumab therapy, a thorough fundus examination, OCT, and fluorescein angiography should be performed to evaluate potential early retinal and choroidal alterations. Regular monitoring, ideally at three- to six-month intervals during treatment, may facilitate early detection of ocular toxicity signs, even in the absence of symptoms, enabling prompt intervention and informed decisions regarding the continuation or cessation of immunomodulatory therapy.

## Conclusion

This case emphasizes the importance of proactive surveillance for ocular toxicities in patients receiving ipilimumab therapy. Early detection and an interdisciplinary approach with the oncology team may improve visual outcomes and provide a safer management strategy for these patients, particularly in a context where immunotherapy represents an increasingly common treatment alternative for advanced melanoma.

## Notes

### Patient consent

The author has explicit consent from the patient, agreeing to the publication in its present form.

### Competing interests

The author declares that he has no competing interests.
